# 396. Incidence and Attributable Mortality of *Clostridioides difficile* Infection Among US Adults 18-64 Years of Age

**DOI:** 10.1093/ofid/ofac492.474

**Published:** 2022-12-15

**Authors:** Holly Yu, Tamuno Alfred, Jingying Zhou, Jennifer Judy, Margaret A A Olsen

**Affiliations:** Pfizer Inc., Collegeville, Pennsylvania; Pfizer Inc, New York, New York; Pfizer Inc, New York, New York; Pfizer, New York, New York; Washington University School of Medicine, St. Louis, Missouri

## Abstract

**Background:**

Studies characterizing the burden of *Clostridioides difficile* infection (CDI) have largely focused on older adults, with limited data among those < 65 years of age insured under commercial plans.

**Methods:**

This retrospective cohort study from 2012–2020 used Optum’s de-identified Clinformatics® Data Mart of about 42 million commercially insured persons. CDI was defined by ICD9/ICD10 diagnosis codes or a combination of CDI diagnosis/testing with antibiotic receipt; cases occurring ≤60 days after prior CDI occurrences were excluded. Annual CDI incidence was evaluated among individuals who were 18–64 years old and enrolled in an Optum commercial plan by January 1 of the corresponding year. Mortality was evaluated in persons with CDI from 2016–2018 who were continuously enrolled in the database for ≥12 months prior; follow-up occurred through the earliest of 12 months, disenrollment, or death. To assess CDI-attributable mortality, CDI+ cases were matched 1:1 to CDI– controls by the propensity score for CDI. Mortality was stratified by age group, acquisition type, and hospitalization status.

**Results:**

CDI incidence was generally stable from 2012–2016 (217–220 and 112–118 episodes per 100,000 person-years (PY) in the 50–64- and 18–49-year age groups, respectively) before decreasing gradually between 2016 and 2020 to 139 episodes per 100,000 PY (50–64-year age group) and to 66 episodes per 100,000 PY (18–49-year age group) (**Figure 1**). In the 50–64-year age group, CDI-attributable mortality increased to 2.2% at 12 months (CDI+, 4.2%; CDI−, 2.0%), with larger attributable differences observed among hospitalized (healthcare associated [HA], 18.1%; community associated [CA], 7.0%) vs nonhospitalized (HA, 3.3%; CA, −0.1%) patients (**Figure 2**). The CDI-attributable mortality rate of 0.6% at 12 months among the 18–49-year age group was lower than that in the 50–64-year age group but trended similarly (CDI+, 1.2%; CDI−, 0.6%).

**Figure:**
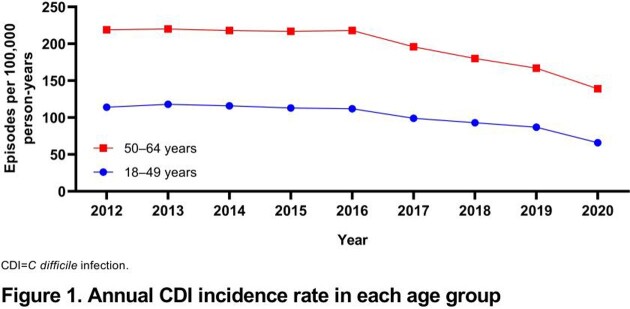

**Figure:**
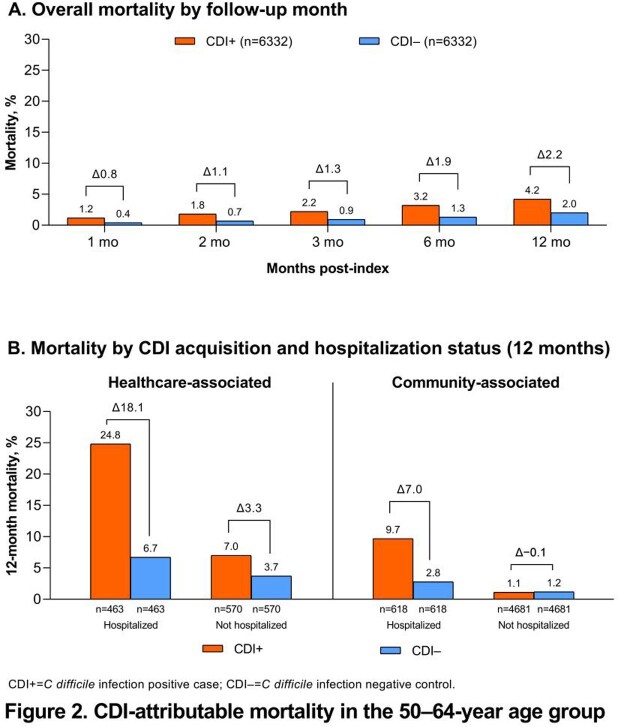

**Conclusion:**

As reported previously, both CDI incidence and attributable mortality among US individuals 18–64 years increased with age. Identifying high-risk groups among non-elderly adults is warranted to develop better strategies for effective prevention.

Funded by Pfizer Inc.

**Disclosures:**

**Holly Yu, MSPH**, Pfizer Inc: Employee|Pfizer Inc: Stocks/Bonds **Tamuno Alfred, PhD**, Pfizer Inc: Employee|Pfizer Inc: Stocks/Bonds **Jingying Zhou, MA**, Pfizer Inc: Employee|Pfizer Inc: Stocks/Bonds **Jennifer Judy, MS, PhD**, Pfizer Inc: Employee|Pfizer Inc: Stocks/Bonds **Margaret A A. Olsen, PhD, MPH**, Pfizer Inc: Advisor/Consultant|Pfizer Inc: Grant/Research Support.

